# Regulating TRAIL Receptor-Induced Cell Death at the Membrane: A Deadly Discussion

**DOI:** 10.2174/157489211796957757

**Published:** 2011-09

**Authors:** Sarah Shirley, Alexandre Morizot, Olivier Micheau

**Affiliations:** 1 INSERM, U866, Dijon, F-21079 France; Faculty of Medicine and Pharmacy, Univ. Bourgogne, Dijon, F-21079 France; 2 Centre Georges-François Leclerc, Dijon, F-21000 France

**Keywords:** Chemotherapy, death domain, death effector domain, DISC, FADD, c-FLIP, scaffold, TRAIL, TRAIL-R4.

## Abstract

The use of TRAIL/APO2L and monoclonal antibodies targeting TRAIL receptors for cancer therapy holds great promise, due to their ability to restore cancer cell sensitivity to apoptosis in association with conventional chemotherapeutic drugs in a large variety of tumors. TRAIL-induced cell death is tightly regulated right from the membrane and at the DISC (Death-Inducing Signaling Complex) level. The following patent and literature review aims to present and highlight recent findings of the deadly discussion that determines tumor cell fate upon TRAIL engagement.

## INTRODUCTION

TRAIL, also known as APO2-L or TNF-Related Apoptosis-Inducing Ligand and its derivatives, including agonistic antibodies targeting TRAIL receptors or PARAs (ProApoptotic Receptor Agonists), are attractive compounds for cancer therapy due to their ability to induce tumor regression without significant side effects [1]. Extensive efforts are being made to evaluate the efficacy and the safety of these combinations in clinical trials [2], and there are many instances in the patent literature of efforts to use polypeptides derived from the TRAIL ligand, [3-10] as therapy against cancerous cells. Other patent applications seek to use agonistic antibodies directed against the TRAIL receptors in order to induce the TRAIL apoptotic pathway [11-19], or TRAIL ligand gene transfer [20]. Amgen has recently published interesting results of a phase Ib study on twenty five patients with advanced nonsquamous non-small-cell lung cancer, treated with recombinant TRAIL (Dulanermin / AMG 951) combined with paclitaxel, carboplatine and Bevacizumab (PCB). Combining Dulanermin with PCB was well tolerated in patients, but importantly was more efficient than PCB alone for first line treatments, with an overall response rate of 58% as compared to 35% for PCB [21]. For a review on current ongoing clinical trials using PARAs see [22].

TRAIL belongs to the TNF (Tumor Necrosis Factor) superfamily of ligands and receptors. Ligands of this family generally recognize and bind to a limited subset of cognate receptors on the cell surface, leading to signal transduction cascades downstream of the receptor, allowing the activation of a large panel of signaling pathways including NF-kB- or caspase-activation. These type I transmembrane proteins contain two to four cysteine-rich domains (CRDs) in their extracellular region, and an intracellular domain that enables the recruitment of adaptor proteins, driving the activation of a particular signaling pathway.

The receptors of this family, which includes TNFR1, CD95/Fas, TRAIL-R1/DR4, TRAILR2/DR5, DR3, and DR6, contain an intracellular stretch of approximately 80 amino acids, called the Death Domain (DD), which is necessary and sufficient for the triggering of the apoptotic programme [23, 24]. With the exception of DR6, whose ligand has only recently been proposed to be a beta-amyloid precursor protein [25], death domain containing receptors are recognized by ligands of the TNF superfamily. These cognate ligands share a common structural motif, the TNF homology domain, which allows their binding to the CRD of TNF receptors [26]. They can be cleaved by metalloproteinases to form soluble cytokines, however, the capacity of the soluble forms of the death ligands to induce apoptosis is significantly lower than the membrane-bound forms [27, 28]. Ligands such as TRAIL, FasL and TNF can, however, be produced as recombinant proteins and used for anticancer therapy [29]. Unlike DR3, whose expression is mainly restricted to T lymphocytes [30], TNFR1, Fas, TRAIL-R1 and TRAIL-R2 were demonstrated to be widely expressed by tumor cells, which prompted the evaluation of their cognate ligands for cancer therapy. TNF and Fas ligand, however, were rapidly shown to be toxic *in vivo*. Their administration triggers fulminant hepatic failure in mice [31], hampering their application for cancer therapy. TRAIL, unlike Fas and TNF, was shown to be safe in experimental animal models [32], as well as in patients, as demonstrated by ongoing clinical trials [33]. Similarly, antibodies targeting agonistic TRAIL receptors, including mapatumumab or lexatumumab, are also well tolerated in patients [33-35]. 

Besides its lack of evident toxicity *in vivo*, TRAIL has gained increasing interest for cancer therapy due to at least four major properties. First of all, TRAIL is naturally involved in tumor metastasis immune surveillance by NK cells [36]. Accordingly, TRAIL-null mice are tumor prone [37] and TRAIL-R-deficient mice exhibit enhanced lymph node metastasis in a model of drug-induced skin carcinogenesis [38]. Second, amongst the ligands of the TNF superfamily, TRAIL is the only member that exhibits a relative selectivity for tumor cells [39, 40]. Hence, it has been demonstrated that while both normal and immortalized cells are resistant to TRAIL-induced apoptosis, Ras- or myc-transformed cells become sensitive [39, 41]. Third, TRAIL-induced cell death is largely independent of p53 [42]. It should be noted however that TRAIL and its receptors are p53 targets [43-46] and that sensitization to TRAIL-induced cell death by chemotherapeutic drugs has sometimes been associated with p53-induced mitochondrial activation either through the activation of Bax [47] or puma [48], as well as through the upregulation of TRAIL-R2 [43, 49] or TRAIL [50]. On the other hand, activation of p53 by some chemotherapeutic drugs may be detrimental to TRAIL-induced apoptosis. Likewise, the combination of TRAIL and oxaliplatin in p53 wt colon carcinoma cell lines was shown to be inefficient due to the p53-dependent up-regulation of TRAIL-R3 [51]. Finally, combinations that associate TRAIL with chemotherapy generally restore tumour cell sensitivity to apoptosis [6, 7, 12], irrespective of TRAIL-R4 expression, or mitochondrial inhibition [52], while having little effect on normal cells [53]. The molecular mechanisms that underlie sensitization to cell death induced by death domain containing receptors encompass a wide panel of events, and depend on both the drug and the cell type [54]. 

At the proximal level, sensitization to TRAIL or Fas ligand was shown to involve receptor up-regulation [55-60], c-FLIP downregulation [42, 61-64], restoration of caspase-8 expression [65, 66] or enhanced DISC formation [67-71]. Downstream of the DISC, sensitization to TRAIL-induced apoptosis was associated with the deregulation of cell survival proteins including, Bcl-2, Bcl-XL, Mcl-1, HSP27, survivin, IAPs [60, 72-75] or pathways such as AKT and NF-kB [76-79].

TRAIL-induced cell death engagement is subject to an exceptional level of control, with many different proteins interacting throughout the apoptotic cascade. The following chapters will focus on the regulation of TRAIL signaling at the membrane and DISC level.

## TRAIL SIGNALING

TRAIL-induced apoptosis involves several major events. The main constituents of the TRAIL receptor DISC and experimental evidence for their presence and function as compared to TNFR1 or Fas are summarized in Table **I** [80-11]. Initiation by ligand binding to the receptors is followed by recruitment of adaptor proteins to the intracellular region of the receptors. The adaptor proteins in turn recruit initiator caspases, forming the Death-Inducing Signaling Complex (DISC), a large macromolecular complex in which caspase-8 and -10 are activated and released for the triggering of apoptosis either directly or indirectly through the mitochondria via the protein Bid Fig. (**1**). 

**Fig. (1):**
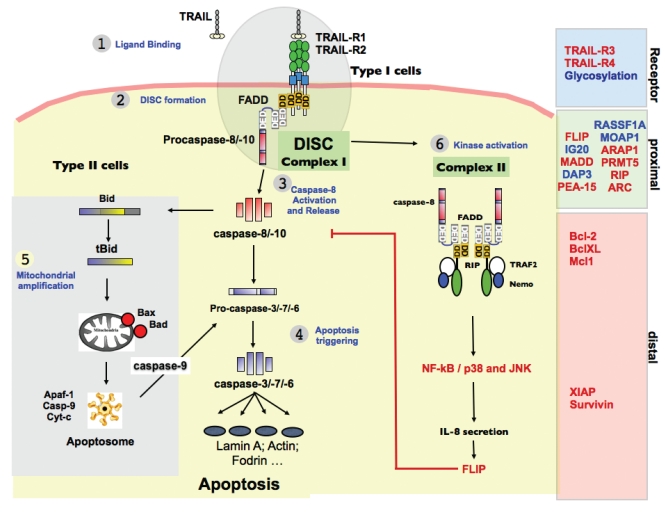
**TRAIL signaling pathway and regulatory proteins.** Binding of the TRAIL ligand to TRAIL-R1 or TRAIL-R2 (1) induces the recruitment of FADD and caspase-8 to these receptors, forming the membrane DISC or complex I (2), in which pro-caspase-8 is activated, leading to the release of the active caspase-8 in the cytosol (3) and allowing the engagement of the apoptotic cascade (4). The mitochondrial amplification loop (5) can be required in some cells to induce the activation of the effector caspase-3. A second complex has recently been described (6), which induces activation of survival signaling pathways leading to transcription factors, which can result in cytokine secretion and increase levels of the inhibitory protein c-FLIP. Regulatory processes of the TRAIL pathway can be divided into three groups: receptor level, proximal, and distal regulation. Inhibitory proteins are shown in red.

TRAIL triggers apoptosis following binding to one of its cognate death receptors, TRAIL-R1 (DR4) and TRAIL-R2 (DR5). Like Fas, but unlike TNFR1[89], TRAIL binding to TRAIL-R1 and TRAIL-R2 induces the formation of the DISC at the membrane level, through homotypic interactions Fig. (1). The DD of the agonistic receptors and that of the adaptor protein FADD allow the recruitment of caspase-8, caspase-10 or c-FLIP through their respective DED (Death effector Domain) [80, 112]. TRAIL-induced cell death can efficiently be regulated at the receptor level by antagonistic receptors [53], at the proximal level by c-FLIP [113] or further downstream by Bcl-2 family members [114] or inhibitors such as XIAP [115, 116] or Survivin [117] Fig. (**1**). 

TRAIL non-apoptotic signaling activities include NF-kB, ERK or p38 activation. A secondary complex, which is not membrane bound (Table **I**), has been proposed to arise sequentially from complex I to trigger MAPK activation [110]. Sequential generation of two distinct functional complexes provide clues to TRAIL's pleiotropic signaling activities, that depending on the cell type, lead to apoptosis, survival or cell differentiation. This secondary complex may explain why, for instance, terminal keratinocyte differentiation induced by TRAIL proceeds both through MAPK and caspase activation [118]. Albeit less characterized (Table **I**), a similar secondary complex may also arise upon Fas stimulation [105]. Keeping in mind that keratinocytes express large amounts of intracellular Fas ligand [119], it could be of interest to define whether this death ligand/receptor set may substitute for TRAIL deficiency to induce cell differentiation, or whether the Fas pathway only affords apoptotic triggering in this cell type. Other differentiation functionalities, associated with MAPK activation have been attributed to TRAIL, including in intestinal cells [120], skeletal myoblasts [121, 122], osteoclasts [123], T helper cells [124] and in dendritic cells [125]. 

Although of great interest, these non-apoptotic features of TRAIL will not be developed any further here. Rather, the following review will mainly focus on TRAIL-induced cell death regulation from complex I.

## CONTROLLING TRAIL-INDUCED CELL DEATH AT THE MEMBRANE LEVEL

### Antagonistic Receptors

TRAIL-R3 (DcR1) and TRAIL-R4 (DcR2) can specifically inhibit TRAIL signaling at the membrane level [45, 126]. These two antagonistic receptors lack a functional DD [53] and impair TRAIL-induced cell death through distinct mechanisms Fig. (**2A**). TRAIL-R3 is devoid of an intracellular domain, but harbours a GPI anchor which drives its expression to lipid rafts [127]. TRAIL-R3 prevents DISC assembly through its ability to compete for TRAIL binding, resulting in the titration of TRAIL within the lipid rafts Fig. (**2B**). TRAIL-R4 on the other hand, is much more similar to TRAIL-R2, and contains an intracellular domain that includes a truncated death domain. Unlike TRAIL-R3, TRAIL-R4 is recruited with TRAIL-R2 within the DISC upon TRAIL engagement, and inhibits initiator caspase activation Fig. (**2C**), probably through steric hindrance [23]. TRAIL-R4 has been shown to form a specific heteromeric complex with TRAIL-R2 through the preligand assembly domain (PLAD), a domain encompassing the first CRD of both receptors but, contrary to our findings, PLAD-mediated TRAIL-R4 and TRAIL-R2 association was suggested to be ligand-independent [128]. The interaction of death-domain containing receptors via the PLAD is proposed to induce a parallel dimeric conformation of the receptors that can account for homotypic as well as heterotypic associations in the absence of ligand Fig. (**2A**). Ligand binding causes a conformational change in the pre-assembled receptor complex that facilitates receptor clustering and DISC formation Fig. (**2A**). 

Similar to TRAIL, some agonistic antibodies are able to engage TRAIL signaling through DISC formation Fig. (**2E**). These antibodies, which selectively target either TRAIL-R1 or TRAIL-R2, efficiently induce cell death in cells that express TRAIL-R3 or TRAIL-R4, unlike TRAIL ligand itself Fig. (**2E**-**F**). Regardless of the stoichiometry of the DISC components, the key common event for the triggering of signaling activity is oligomerization, which allows neighbouring initiator caspases to form specific activating dimers Fig. (**2D**). A proximity-induced dimerization model was proposed to explain the activation of caspase-8 [129]. Recently, a very elegant approach of reconstitution of the Fas DISC using recombinant proteins, revealed a two-step activation mechanism involving both dimerization and proteolytic cleavage of procaspase-8 as obligatory steps for death-receptor-induced apoptosis [130]. Little is known about the stoichiometry of this scaffold. The DD and the DED, like the caspase recruitment domain (CARD) family or the pyrin domain (PYD), share a six-helical bundle structural fold feature that accounts for protein-protein interaction, the arrangement of which defines the stoichiometry of the multimolecular scaffold to which they are recruited. A crystal structure of RAIDD and PIDD, two DD-containing proteins, which are not required for TRAIL signaling but are closely related to the adaptor protein FADD, revealed an asymmetric core complex comprised of seven RAIDD DDs and five PIDD DDs assembled through 3 major interfaces [131]. More recently, the crystal structure of FADD and Fas was obtained, unveiling a tetrameric arrangement [132]. The crystal structure of FADD and TRAIL-R2 or TRAIL-R1 is not known for the moment but, assuming that the assembly of the TRAIL DISC mimics that of Fas, the formation of heteromers of TRAIL-R2 and TRAIL-R4 is likely to disturb the highly ordered arrangement that accounts for caspase-8 activation within the TRAIL DISC since the truncated death domain of TRAIL-R4 is unable to interact with FADD and thus cannot recruit a caspase-8 monomer. According to this hypothesis, we propose a model of DISC arrangement disruption by TRAIL-R4 as compared to the arrangement of a DISC composed of TRAIL-R2 and TRAIL-R1 Fig. (**2D**). In the latter complex, each receptor recruits an initiator caspase, the proximity of which is favourable for a full activation of caspase-8. Recruitment of TRAIL-R4 within the TRAIL DISC, however, alters caspase-8 dimer formation and therefore inhibits caspase-8 activation within the DISC Fig. (**2D**). Arrangement of the DISC and, in particular, caspase-8 proximity is a limiting step for the initiation of the apoptotic signal. Accordingly, it has been demonstrated that enforced ligand covalent trimerization accelerates TRAIL-induced caspase-8 activation and cell death [133]. In line with the requirement of these adaptor proteins to build a proper scaffold for caspase-8 activation, US6015712 raises the possibility of inhibiting FADD expression for therapeutic intervention related to diseases in which the death signaling pathway is activated inappropriately [134]. 

## LIPID RAFTS AND TRAIL

Death domain-containing receptors of the TNF superfamily have a tendency to self-aggregate, owing to their DD. Their overexpression triggers apoptosis [135]. Therefore, it may be assumed that initiation of DISC formation is tightly controlled at the membrane level. The Silencer Of Death Domain (SODD), a DD containing protein identified by yeast two hybrid assay to interact with TNFR1, was proposed to prevent constitutive signaling of tumor necrosis factor receptor 1 (TNFR1) in the absence of TNFα [136]. Generation of mice deficient for SODD, however, failed to support a function regarding the control of TNFR1 aggregation [137]. At the moment it is not clear how these receptors are maintained in an inactive state at the membrane. Membrane lipid composition and fluidity could take part in avoiding receptor self-association. Supporting this hypothesis, ionizing radiation and UV rays [138, 139], which are known to change membrane fluidity, as does cholesterol depletion, induce Fas receptor clustering on the cell surface independently of FasL [140-142]. Alternatively, DISC formation in lipid rafts may account for efficient Fas-induced apoptosis triggering [143]. Cholesterol enriched membranes, however, have not been associated with TRAIL-DISC formation [144-148] with the exception of one study in which TRAIL-DISC formation in lipid rafts was clearly demonstrated [149]. Another indication suggesting that TRAIL signaling might occur within the cholesterol rich membrane domains was the discovery that palmitoylation is required to target TRAIL-R1 to lipid rafts [146]. In a mechanism similar to that of Fas, palmitoylation of TRAIL-R1 was shown to be required for redistribution of actin cytoskeleton-linked rafts, receptor oligomerization and cell death. However it was also found that TRAIL-R2 was not palmitoylated. Although some TRAIL DISC components can be found within lipid rafts [149], thus far it has not been clearly demonstrated that caspase-8 activation occurs within these structures. In addition, with the exception of TRAIL-R3, which localizes readily within lipid rafts, TRAIL-R1, TRAIL-R2 and TRAIL-R4 are mainly expressed in non-lipid raft-containing membranes at the steady state, where most DISC analysis assays demonstrate capase-8 activation upon TRAIL stimulation [23]. In line with these findings, it should be noted that edelfosine-induced cell death requires Fas translocation and aggregation within lipid rafts, but not TRAIL receptors [150]. 

## O-GLYCOSYLATION

An additional level of complexity regarding the regulation of TRAIL signaling was recently found, following the discovery that O-glycosylation of TRAIL-R1 and TRAIL-R2 is a prerequisite for DISC formation and apoptotic triggering [151]. The finding that O-glycosylation controls cell sensitivity to TRAIL-induced cell death could be an important finding, as alterations in glycosylation profiles are often found in cancer patients [152] and during cancer progression [153]. Of particular interest are the findings that in normal human mammary epithelial cells, RAS-induced transformation triggers drastic changes in the glycosylation profile of cell surface proteins [154], and enhances TRAIL DISC formation and caspase-8 activation upon TRAIL stimulation [39]. It remains, however, to be determined whether these changes are sufficient to account for TRAIL tumor cell selectivity.

## RECEPTOR TURN-OVER/TRAFFICKING

Little is known about TRAIL receptor trafficking, yet the first requirement to engage TRAIL-induced cell death is the availability of TRAIL agonistic receptors at the cell surface. Epigenetic dysregulation of TRAIL antagonistic receptors, TRAIL-R3 and TRAIL-R4, or of TRAIL agonistic receptors TRAIL-R1 and TRAIL-R2, has been documented to varying extents [155-157], leading to the loss of expression of the receptors in tumor cells and giving rise to resistance to TRAIL-induced cell death [158]. Recently, a yeast two-hybrid screen uncovered ARAP1, an ArfGAP and RhoGAP adapter protein, as a TRAIL-R1-binding partner. ARAP1 was shown to bind to TRAIL-R1 and TRAIL-R2 in co-expression experiments, but was unable to interact with DR6, another death-domain containing receptor. At the endogenous level, ARAP1 interacted with TRAIL-R1 in a TRAIL- and time-dependent manner. Downregulation of ARAP1 induced a loss of membrane expression of TRAIL-R1 and partly impaired TRAIL-induced cell death [159]. Since ARAP-1 was shown to regulate EGFR endocytosis [160], it was proposed that ARAP1 could play a role in regulating TRAIL-R1 trafficking and thus TRAIL-induced signaling.

## CONTROLLING TRAIL-INDUCED CELL DEATH AT THE DISC LEVEL

The stoichiometry and composition of the TRAIL DISC is not clearly defined, but important regulatory proteins are proposed to be involved in the regulation of TRAIL signaling, owing to their ability to be recruited to the DISC or to interfere with proteins participating in TRAIL DISC formation.

## c-FLIP

Cellular FLIP is probably the most important inhibitor of receptors containing death domains. Three main isoforms of c-FLIP are expressed in human cells: c-FLIP_L_, c-FLIP_S_, and c-FLIP_R_. Depending on the cell line and on the levels of FLIP expression, all three proteins can be found within the Fas DISC due to their N-terminal domain which contains two DED repeats similar to caspase-8 or caspase-10 [54]. The contribution of c-FLIP_R_ regarding the control of TRAIL-induced cell death has not yet been characterised. The short isoforms of c-FLIP, c-FLIP_S_ and c-FLIP_R_ both possess a truncated C-terminus. In addition to the tandem DED repeat, c-FLIP_L _harbours an extended C-terminal domain that is structurally similar to procaspase-8, but is devoid of an active catalytic domain. The cysteine residue that is normally required for caspase-8 function is replaced by a tyrosine that renders c-FLIP_L_ inactive [113]. These isoforms of c-FLIP, although generally expressed at a lower level compared to caspase-8 in most tumor cell lines, are recruited within the DISC together with caspase-8 where they inhibit the activation of the initiator caspases, impairing apoptotic triggering. The molecular mechanisms by which the long and the short FLIP isoforms inhibit TRAIL-induced cell death differ substantially. In the absence of c-FLIP, caspase-8 is activated in two-steps: dimerization, followed by cleavage [130]. One caspase-8 molecule brought in close proximity within the DISC to another caspase-8 can cleave itself and the other caspase-8 to induce the release of the catalytic subunits, p10 and p20, which form the mature caspase-8 that initiates the triggering of apoptosis. In the presence of c-FLIP_S_, procaspase-8 remains inactive within the DISC and the cells survive. Heterodimerization of c-FLIP_L_ with procaspase-8 within the DISC, however, mimics procaspase-8 dimerization and leads to caspase-8 activation in the absence of procaspase-8 cleavage [161]. Caspase-8 is maintained within the DISC and cannot be released to the cytosol because the generation of the p20 subunit of caspase-8 cannot occur in the presence of c-FLIP_L_. Active caspase-8 therefore remains sequestered within the DISC, where it can still induce the cleavage of a number of substrates including c-FLIP, RIP and as yet to be discovered unidentified proteins, recruited within the DISC or in close proximity [130, 161]. While all isoforms of c-FLIP efficiently inhibit Fas ligand- and TRAIL-induced cell death, subcellular confinement of active caspase-8 is only asscociated with c- FLIP_L _so far. The finding that c-FLIP_L_ induces caspase-8 activation within the DISC represents another degree of control regarding the regulation of TRAIL signaling. RIP cleavage at the DISC level in these circumstances could play a role in controlling TRAIL-induced necrosis [162], NF-kB activation [163-165] or other non-apoptotic functions. The possibility of using of RNA interference to inhibit cFLIP to circumvent TRAIL resistance has been proposed in the patent application US20040126791 [166]. 

## MADD-IG20

The MAPK-Activating Death Domain (MADD) variant, also coined Rab3-GAP, which is constitutively expressed in many cancer cells [167], was the first member of the family found to harbour a low homology DD and to interact with TNFR1 [168]. All of these splice variants contain a DD, but their contribution to the regulation of death receptor differs. IG20 was found to interact with both TRAIL-R1 and TRAIL-R2 and to enhance TRAIL DISC formation, thus increasing TRAIL-induced cell death Fig. (**3A**) [169]. MADD on the other hand, albeit structurally close to IG20 since both isoforms contain a DD and a leucine zipper domain, was demonstrated to behave as a negative regulator of TRAIL [169]. Similar to IG20, MADD was shown to interact with TRAIL-R1, but its expression was suggested to impair TRAIL DISC formation through the inhibition of caspase-8 recruitment Fig. (**3A**), leading to survival of the cells expressing MADD. Modulation of MADD to overcome resistance to TRAIL has been suggested as a possible therapy [170].

## PRMT5

The protein arginine methyltransferase 5 (PRMT5) was found to interact with TRAIL-R1 by a proteomic screen [171]. Coexpression experiments revealed that PRMT5 could also interact with TRAIL-R2 but not other receptors of the TNF family, including Fas or TNFR1 Fig. (**3B**). Knockdown of PRMT5 was shown to sensitize tumor cells to TRAIL-induced cell death, while PRMT5 overexpression conferred TRAIL resistance [171]. PRMT5-mediated TRAIL resistance required NF-kB activation but was demonstrated to be methyl transferase-independent [171]. It is unclear for the moment how PRMT5 binds to TRAIL-R1. PRMT5, besides its methyl transferase activity, has no DD and no DED. Like IG20 and MADD, recruitment of PRMT5 to TRAIL-R1 appeared to be ligand independent. Finally, PRMT5 inhibitory function was suggested to occur regardless of TRAIL DISC formation. 

## DAP3

DAP3 (Death Associated Protein 3) is a GTP-binding adapter protein that interacts directly with the FADD DED Fig. (**3C**). Though the predominant function of DAP3 concerns maintenance of mitochondrial function, it is also able to influence apoptotic signaling [172]. Its recruitment to the TRAIL DISC was shown to regulate caspase-8 activation in a GTP-dependent fashion [173], so it was therefore proposed that DAP3 could be a direct regulator of TRAIL-induced caspase-8 activation and cell death Fig. (**3C**). It was later found that DAP3 is a ribosomal protein that is mainly localized to the mitochondrial matrix, and which cannot interact with FADD unless subcellular compartments are compromised [174, 175]. While the relative expression level of DAP3 in the cytosolic fraction remains unclear, targeted gene inactivation of dap3 confirmed the regulatory function of this GTP-binding adapter protein regarding TRAIL-induced cell death in particular, but also apoptosis induced by death receptors including TNFR1 or Fas [172]. Inactivation of dap3, however, had little or no impact on apoptosis induced by staurosporine or etoposide, two chemotherapeutic compounds known to target the intrinsic mitochondrial pathway. The question as to how DAP3 controls caspase-8 activation within the TRAIL DISC remains open. The master kinase STK11 was suggested to play a significant regulatory function upstream of DAP3 in osteosarcomas. In this study, STK11 was found to interact with DAP3 and enhance TRAIL-induced cell death through its serine/threonine kinase activity [176]. 

## PEA15/PED

Phosphoprotein enriched in astrocytes (PEA15, also known as PED or HTMA), is a small protein (15kDa) composed of a N-terminal DED and a C-terminal tail of irregular structure. PEA15 was first reported to inhibit apoptosis induced by Fas and TNFR1 [177] and later found to be recruited to the TRAIL DISC, and to inhibit TRAIL-induced cell death, thus accounting for cell resistance in gliomas [178]. PEA15 is an endogenous substrate of kinases including PKC, Akt and CAMKII, and phosphorylation of PEA-15 on the serine 116 promotes FADD-binding [179]. It has been demonstrated recently that PEA-15 could promote mitochondrial-dependent type II Fas-induced cell death in cells inactivated for PTEN [180]. It is reported, however, that Akt-mediated phosphorylation of PEA-15 on the serine 116 residue impairs Fas DISC formation through the sequestration of FADD and not through the recruitment of PEA-15 within the Fas DISC. However when PTEN is functional, Akt is inactivated, serine 116 of PEA-15 is not phosphorylated and PEA-15 is unable to interact with FADD, allowing FADD recruitment, TRAIL DISC formation and apoptosis after TRAIL stimulation Fig. (**3D**). Interestingly, TPA, a phorbol ester also coined PMA, which has been known for a long time to inhibit Fas and TRAIL-induced apoptosis [181], and to impair DISC formation [182, 183], upregulates PEA-15 expression and enhances PEA-15 phosphorylation at serine 116 [184]. 

## RASSF1A/MOAP1

The RAS association domain family 1A (RASSF1A) protein, although devoid of any characterized DD or DED, is a tumor suppressor that is shown to interact with the DD of TNFR1 and TRAIL-R1 [185]. RASSF1A links death receptors to the mitochondrial pathway through the protein modulator of apoptosis 1 (MOAP-1). Ectopic expression of RASSF1A enhances death receptor induced cell death while downregulation of RASSF1A or MOAP-1 inhibits bax activation and cytochrome c release [186]. This adaptor protein is found in an inactive state in the cytoplasm, and is activated upon recruitment with RASSF1A within the TRAIL DISC upon stimulation Fig. (**3E**). Release of MOAP-1 from the DISC is proposed to induce a conformational change that allows Bax recruitment and activation, leading to the activation of the mitochondrial amplification loop that sustains caspase activation and apoptosis [185]. Given that epigenetic-driven loss of RASSF1A protein expression is often observed in tumors of higher grade [187] and that RASSF1A is a potential regulator of TRAIL, demethylation agents could prove useful to restore TRAIL sensitivity in high grade tumors.

## OTHER DD- OR DED-CONTAINING ADAPTOR PROTEINS INVOLVED IN THE REGULATION OF TRAIL AT THE DISC LEVEL (DEDD, RIP, TRADD, ARC)

All DD-containing proteins may potentially regulate TRAIL-induced apoptosis owing to their ability to induce homotypic interactions with TRAIL-R1, TRAIL-R2 or FADD. Likewise, RIP and TRADD contain a DD and are likely to participate in the TRAIL DISC. However, so far their recruitment has been found to be cell dependent, as neither TRADD nor RIP are recruited to the DISC in BJAB cells, a B lymphoma [188] while RIP is shown to be recruited in colon cancer cell lines [189]. Similar to TNFR1 [89], TRAIL induces the sequential formation of two distinct complexes Fig. (**1**). 

TRAIL complex I, corresponds to the DISC. Complex I is localized at the cell membrane and is essential for caspase-8 activation while Complex II, a complex arising from the membrane TRAIL DISC, has been proposed to activate survival pathways due to the integration of several adaptor proteins including TRADD and RIP [110]. RIP recruitment at the cell membrane can occur in the absence of FADD or TRADD in the TNFRI complex I, as well as independently of FADD in the Fas DISC [85, 162]. Therefore, it is likely that the recruitment of RIP to the TRAIL DISC or to complex II is independent of these adaptor proteins. However, indirect interactions cannot be excluded despite the finding that RIP and TRADD are able to interact directly with FADD and some death receptors in overexpression experiments [164]. In addition to its DD, RIP contains a kinase domain whose activity is required for TRAIL-induced necrosis, but is compulsory for NF-kB activation upon TRAIL stimulation [162]. Though RIP itself is thought to be essential to trigger NF-kB activation upon TNF stimulation [163], it has recently been demonstrated that RIP is in fact not essential for TNFR1-induced NF-kB activation [190]. Whether this holds true for TRAIL remains to be determined. Inhibition of RIP expression, nonetheless, promotes TRAIL-induced cell death [191, 192]. 

Similar to DD-containing proteins, proteins that harbour a DED such as DEDD or DEDD2, are capable of interfering with known DISC components, including c-FLIP or caspase-8. Accordingly, DEDD2, a DED-containing protein that exhibits a close sequence homology with DEDD, was shown to interact with c-FLIP and to enhance apoptosis induced by Fas and TRAIL [193]. However, DEDD2 was unable to interact with FADD or caspase-8, even though DEDD2-mediated sensitization to apoptosis was restricted to Fas and TRAIL. Its overexpression failed to enhance staurosporine- or bax-induced cell death.

DD- and DED-containing proteins feature a 6-alpha-helical bundle structure fold that mediates dimerization by electrostatic interactions. It is generally accepted that homotypic interactions occur between similar domains, but unconventional heterotypic interactions may also account for the regulation of TRAIL signaling. Accordingly, Arc a protein containing a CARD domain, which is a protein interaction module similar to the DD or the DED, was found to interfere with death receptor induced cell death, owing to its ability to bind to FADD and to inhibit DISC formation [194]. 

## CURRENT & FUTURE DEVELOPMENTS

One important bottle neck for TRAIL signaling is probably the engagement of the apoptotic signaling complex from the membrane. Since this signaling pathway seems primarily dedicated to cell killing *in vivo*, TRAIL signaling has to be tightly controlled at the cell surface. This control can be specifically acheived by TRAIL antagonistic receptors but also less selectively by c-FLIP, both of which are found to be conserved throughout evolution. Plasma membrane lipid composition, although not specific to TRAIL, is also likely to play an important role in controlling DISC formation and apopotosis triggering (see Segui and Dimanche-Boitrel, this issue). Moreover, since the discovery of the membrane-bound DISC, composed of the receptor, the adaptor protein FADD, the initiator caspases-10 and -8 and their inhibitor c-FLIP, a plethora of binding partners have been shown to contribute to the regulation of the deadly signal. These proteins act both at the membrane level or at the proximal level due to their ability to interact with DISC or secondary complex components. Regulation of caspase-8 activation from the DISC and initiation of apoptosis is associated with either the disruption or the enhancement of TRAIL-induced scaffold complexes, as well as with the regulation of the mitochondrial pathway. There is an incredible diversity of interacting molecules that have been described so far to take part in the regulation of TRAIL-signaling which add another level of complexity to our understanding of the TRAIL Discussion. Current developments for targeting the TRAIL pathway show promise for cancer therapies, but a deeper understanding of the TRAIL DISC composition and stoichiometry will be crucial for the development of effective TRAIL-based therapeutic approaches. 

## Figures and Tables

**Fig. (2):**
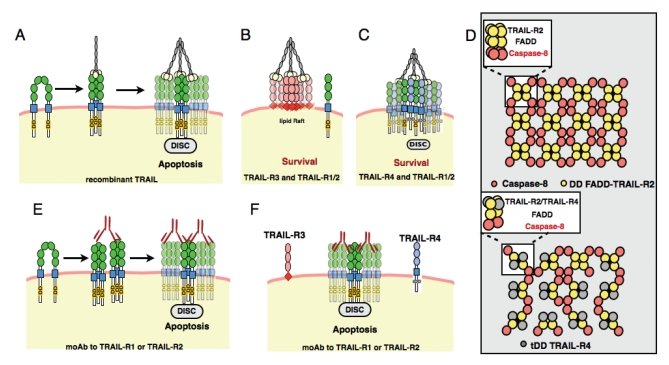
**TRAIL signaling at the receptor level. A, TRAIL ligand** induces aggregation of the TRAIL receptor at the membrane and activates the apoptotic cascade. **B, TRAIL-R3** competes for TRAIL binding, sequestering TRAIL in lipid rafts. **C, TRAIL-R4** forms heteromeric complexes with TRAIL-R2, inhibiting caspase-8 activation. **D, Tentative model** of inhibition of caspase-8 activation by TRAIL-R4. Upon engagement of TRAIL, the receptors aggregate into a highly regular array, whose minimal arrangement is represented as a side view in the white square. This tetrameric interaction module is composed of FADD-TRAIL-R2/Caspase-8 (Yellow and Red circles). Modular arrangement of this module into a platform enhances the proximity-induced dimerization and activation of caspase-8. Recruitment of TRAILR4 (lower panel, grey circles) disrupts caspase-8 arrangement, and thus limits caspase-8 activation. **E-F**, receptor specific agonistic antibodies engage TRAIL DISC, irrespective of TRAIL-R3 or TRAIL-R4 expression.

**Fig. (3):**
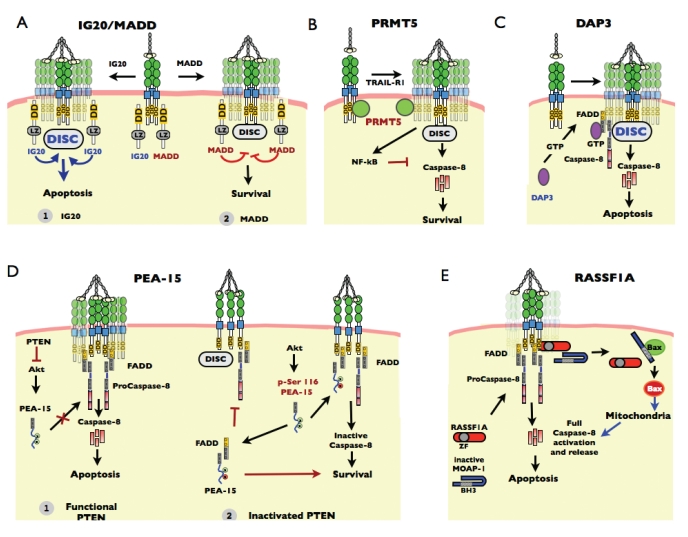
**Mechanisms of TRAIL signaling regulation by different proteins.** See text for details.

**Table I:** Components of the Death Inducing Signaling Complex for Fas, TRAIL and TNF. *Membrane Bound Complex. Main Evidences from native Immunoprecipitation Experiments or from Yeast Two-hybrid and Co-immunoprecipitation Assays.

	TNF-R1	TRAIL-R1, TRAIL-R2	Fas

Apoptosis	**Complex II**	Complex I*	Complex I*
	**(in absence of TNF-R1)**	FADD [80,81]	FADD [82,87,111]
	RIP [83,85,89]	Caspase-8 [80,92]	Caspase-8 [86]
	TRADD [83,85,89]	Caspase-10 [88,93,96]	Caspase-10 [88,90,93,95]
	FADD [83,85,89]	c-FLIP [84,94,97]	c-FLIP [84,91,94]
	Caspase-8 [83,85,89]		
	Caspase-10 [89]		
	c-FLIP [89]		

Non-apoptotic signalling	Complex I *	**Complex II**	**Complex II**
	TRADD [102-104]	RIP-1 [85,110]	**(in absence of CD95)**
	TRAF-2 [102,104]	TRAF2 [110]	FADD [105]
	RIP1 [102]	Caspase-8 [110]	Caspase-8 [105]
	IKKγ [98]	FADD [110]	cFLIP [105]
	IKKα, IKKβ [98,99]	IKKγ [85,110]	RIP1 [100]
	cIAP1 [106-108]	TRADD [85]	
	cIAP2 [106,107]		
	LUBAC ligase complex [101,109]		
